# Cross‐sectional HIV and HCV cascades of care across the regions of Ukraine between 2019 and 2020: findings from the CARE cohort

**DOI:** 10.1002/jia2.26166

**Published:** 2023-09-13

**Authors:** Olga Fursa, Joanne Reekie, Ihor Kuzin, Larysa Hetman, Alina Kryshchuk, Olena Starychenko, Nana Hrytsaiuk, Inna Khodus, Alla Nyzhnyk, Viktoriia Rakhuba, Maryna Kovalevska, Tetiana Maistat, Iryna Pryhoda, Marianna Ahieieva, Olena Varvarovska, Olena Valdenmaiier, Jens Lundgren, Lars Peters, the CARE study group

**Affiliations:** ^1^ Centre of Excellence for Health, Immunity and Infections Rigshospitalet Copenhagen Denmark; ^2^ Public Health Center of the Ministry of Health of Ukraine Kyiv Ukraine; ^3^ Kyiv City AIDS Prevention and Control Center (Kyiv City Clinical Hospital №5) Kyiv Ukraine; ^4^ Kyiv Regional Center for Public Health Kyiv Ukraine; ^5^ Regional Clinical Center for AIDS Prevention and Control of Kharkiv Regional Council Kharkiv Ukraine; ^6^ Mariupol City Hospital №4 named after I.K. Matsuka Mariupol Ukraine; ^7^ Odesa City AIDS Prevention and Control Center Odesa Ukraine; ^8^ Regional Medical Specialized Center of Zhytomyr Regional Council Zhytomyr Ukraine

**Keywords:** HIV, hepatitis C, HIV/HCV, cascade of care, Ukraine, Eastern Europe

## Abstract

**Introduction:**

Eastern Europe is facing major HIV and hepatitis C (HCV) epidemics, with many people living with HIV (PLHIV) and HIV/HCV coinfection living in Ukraine. Despite the previous progress towards care quality improvement, the ongoing war in Ukraine is disrupting HIV and HCV care.

**Methods:**

We described an HIV cascade of care (CoC) in PLHIV from two clinical sites and an HCV CoC for anti‐HCV‐positive PLHIV from six sites in Ukraine, enrolled in the CARE cohort between 1 January 2019 and 1 June 2020. The cross‐sectional HIV CoC and HCV CoC are described at study enrolment.

**Results:**

Of 1028 PLHIV, 1014 (98.6%, 95% confidence interval [CI] 97.7–99.3) were on antiretroviral therapy (ART), and 876 (86.4% of those on ART, 95% CI 84.1–88.4) were virologically suppressed. Of 894 participants on ART >6 months, 90.8% (95% CI 88.7–92.6) were virologically suppressed (HIV‐RNA <200 copies/ml). Of 2040 anti‐HCV‐positive PLHIV, 417 (20.4%, 95% CI 18.7–22.3) were ever tested for HCV‐RNA prior to enrolment, ranging from 4.9% to 54.4% across sites, and 13.5% were currently HCV‐RNA positive. One hundred and eighteen persons (7.3% of ever chronically infected) had received HCV treatment, and 25 persons (1.6% of ever chronically infected) were cured, with variations across sites (0%–7.5%). The site diagnosing 54.4% of people with chronic HCV was the only one providing free RNA testing for all anti‐HCV‐positive persons, while the intra‐country differences in treatment coverage were driven by the number of available direct‐acting antiviral (DAA) courses.

**Conclusions:**

Over 98% of PLHIV in care in both CARE sites in Ukraine were receiving ART, and the target of 90% virally suppressed was achieved in persons >6 months on ART. Only one of six HIV/HCV study sites tested over 50% anti‐HCV‐positive PLHIV for HCV‐RNA and treated over 25% of eligible persons. While free HCV‐RNA testing and DAA treatment are paramount to achieving HCV elimination targets, they remained a challenge in Ukraine in 2019–2020. The extent of the HIV and HCV care disruption during the war will be further assessed in the CARE cohort and compared with the pre‐war findings.

## 1 INTRODUCTION

Ukraine, a country with the second‐largest HIV epidemic in Eastern Europe, was home to an estimated 260,000 people living with HIV (PLHIV) in 2021 [1], of whom, 150,005 were registered in healthcare facilities in Ukraine, excluding non‐government‐controlled areas [2]. While Ukraine has made vast progress towards the UNAIDS 90‐90‐90 targets, the target of 90% people aware of their HIV status being on antiretroviral therapy (ART) had not been met by 2020 [3, 4]. The challenges of providing ART in Ukraine included stigma and discrimination that lead to delayed treatment start or discontinuation [5].

The estimated prevalence of chronic viral hepatitis C (HCV) in the general population in Ukraine was 3.6% in 2018 [6], and the prevalence of HCV coinfection in people newly diagnosed with HIV was found to be 36.3% in 2015 [7]. Following the WHO Global Health Sector Strategy on viral hepatitis [8], in November 2019, Ukraine approved the national strategy to identify and treat 90% of persons with HCV by 2030 [9]. However, despite the availability of affordable direct‐acting antivirals (DAAs) to treat HCV in Ukraine [10], only 15.5% of people diagnosed with HIV/HCV coinfection in 2021 started treatment [11]. Further, no state funding is available for HCV diagnosis, and testing costs are often covered by patients themselves [11].

In addition to the existing challenges, the armed conflict in Eastern Ukraine and the annexation of Crimea has disrupted healthcare services in these areas [12], including a ban on opioid substitution therapy provision [13, 14], complicating ART delivery [15], and limiting harm reduction services and HIV testing [16]. From 24 February 2022, Ukraine is suffering from the massive Russian invasion that threatens its HIV and HCV response [17]. While 12,212 new HIV infections have been registered in 2022, the percentage of PLHIV on ART of those registered in clinical care dropped from 86.6% in 2021 [2, 18] to 76.9% in 2022 [19, 20].

To identify gaps in the HIV and HCV care in clinical settings in Ukraine prior to the war, we constructed HIV and HCV Cascades of Care (CoC) for PLHIV enrolled in the **C** ommon **A** ction against HIV/TB/HCV across **R** egions of **E** urope (acronym: CARE) project.

## 2 METHODS

CARE (https://www.careresearch.eu) was a Horizon2020 project aiming to foster research collaboration between the EU and Eastern Europe. The CARE cohort enrolled 4035 randomly selected PLHIV ≥18 years from Georgia, Ukraine and Russia in the HIV host genetic study, and 3342 HIV/HCV coinfected participants ≥18 years from Ukraine and Russia in the HIV/HCV outcomes study. More details are provided in the CARE study protocol [21].

### 2.1 Ethical considerations

The CARE study was conducted according to the Declaration of Helsinki [22] and followed the requirements of Good Clinical Practice [23]. The study has been approved by the Institutional Review Board of the Public Health Center of the Ministry of Health in Ukraine and reviewed by the External Ethics Review Board. Informed consent for the collection of the clinical data and blood samples was obtained from participants when required by national regulations. All personal data were pseudonymized.

### 2.2 Study population

This paper describes baseline characteristics and presents cross‐sectional HIV and HCV CoCs for participants of HIV and HIV/HCV studies, who were enrolled in Ukraine between 1 January 2019 and 1 June 2020. HIV study participants were enrolled in two HIV clinics from Kyiv and Kyiv Region, while HIV/HCV study enrolled participants in six HIV clinics from Kyiv, Kyiv Region, Kharkiv, Mariupol, Odesa and Zhytomyr. Baseline was the date of study enrolment visit.

### 2.3 HIV CoC

We constructed the cross‐sectional HIV CoC to assess the proportion of participants on ART among those in care, and virologically suppressed among those on ART.

All HIV study participants were considered “*in care*”; PLHIV who received at least one antiretroviral drug at their CARE enrolment visit were considered “*on ART*”; and PLHIV with the most recent HIV‐RNA test result within 12 months before enrolment below 200 copies/ml were considered “*virologically suppressed* . *”* Participants with no eligible HIV‐RNA measurement were assumed to be unsuppressed. We also assessed viral suppression in the subgroup of PLHIV who had been on ART for >6 months before enrolment.

### 2.4 HCV CoC

The cross‐sectional HCV CoC followed the definitions for the HCV CoC stages used in the EuroSIDA study [24] (Table 1).

**Table 1:** Definitions of HCV cascade of care stages evaluated at study enrolment.

Stage number	Stage name	Definition of the stage
**Diagnostic stages**		
1	Anti‐HCV positive	Positive anti‐HCV IgG test result, or a positive HCV‐RNA test result, or data on HCV genotype, or HCV treatment record before or on the enrolment date
2	Ever HCV‐RNA tested	At least one HCV‐RNA test or HCV genotype test performed before or on the enrolment date
3	Currently HCV‐RNA positive	Most recent HCV‐RNA test before or on the enrolment date was positive, or HCV genotyped but not treated on or before or on the enrolment date
**Treatment stages**		
4a	Ever chronically infected with HCV	Estimated number of persons with chronic HCV among all anti‐HCV positive, defined as 79% of antibody positive (based on the serum HCV‐RNA positivity rate in this region in the EuroSIDA study [25])^a^
4b	Ever diagnosed with chronic HCV	Positive HCV‐RNA result, or HCV genotype test, or HCV treatment record on or before the enrolment date
5	Ever started treatment	HCV treatment initiation on or before the enrolment date
6	Treatment completed	Completion of HCV treatment on or before the enrolment date
7	Sufficient follow‐up after treatment completion	Completion of DAA treatment >12 weeks prior to enrolment or completion of IFN treatment >24 weeks prior to enrolment
8	Follow‐up HCV‐RNA available	HCV‐RNA test >12 or 24 weeks after completing treatment (for IFN‐free and IFN‐based therapy, respectively) on or before the enrolment date
9	Cured	The first HCV‐RNA test at least 12 or 24 weeks after the end of last treatment (for IFN‐free and IFN‐based therapy, respectively) and on or before the enrolment date was negative

^a^ The estimated number of ever chronically infected was used as the denominator for the treatment part of the CoC instead of the exact number of diagnosed with chronic HCV, as the latter largely depends on the HCV‐RNA testing coverage.

A bilingual survey on HCV‐RNA testing and treatment availability was completed by six HIV/HCV study investigators, one from each site.

Descriptive statistics were summarized as frequencies and proportions with χ2 *p*‐values for categorical variables. For continuous variables, data were described using the Kruskal−Wallis test and presented as medians and interquartile ranges.

All analyses were performed using SAS 9.4 software (version 9.4; SAS Institute).

## 3 RESULTS

A total of 1028 PLHIV were included in the HIV CoC and 2040 in the HCV CoC, of them, 388 participants were included in both CoC (Table 2). Participants’ baseline characteristics differed between the two studies, especially in the reported HIV transmission mode (percentage of people who inject drugs in the HIV study: 30.2%, HIV/HCV study: 70.6%) and HIV‐RNA (below 200 copies/ml: 85.7% in the HIV study and 66.8% in the HIV/HCV study; not measured in the last year: 4.7% in the HIV study and 20.4% in the HIV/HCV study).

**Table 2:** Baseline characteristics of the sub‐cohorts of PLHIV in the HIV study and HIV/HCV study in the CARE cohort in Ukraine.

Variables		HIV study		HIV/HCV study	
		*N*	%	*N*	%
Number of participants		1028	100	2040	100
Gender	Male	602	58.6	1464	71.8
Ethnicity	White	1027	99.9	2039	99.9
HIV transmission route	PWID	310	30.2	1441	70.6
	Heterosexual	579	56.3	570	28.0
	MSM	134	13.0	25	1.2
ART status	Ever received ART	1023	99.5	2021	99.1
	Currently on ART	1014	98.6	1988	97.5
HIV viral load	<200 copies/ml	882	85.7	1363	66.8
	Not measured in the last 12 months	48	4.7	416	20.4
Prior AIDS diagnosis		369	37.0	976	47.8
HBV coinfection	HBsAg +	55	5.3	239	11.7
HCV coinfection	Anti‐HCV IgG+	383	37.3	2040	100
TB coinfection	Ever diagnosed with TB	189	18.4	588	28.8
Median age, years		39 (35–44)		41 (37–45)	
Median nadir CD4 cell count, cells/μl		201 (70–351)		191 (78–331)	
Median latest CD4 cell count, cells/μl		433 (193–624)		395 (218–585)	

Abbreviations: ART, antiretroviral therapy; HBV, hepatitis B virus; HBsAg+, positive for hepatitis B surface antigen; HCV, hepatitis C virus; MSM, men who have sex with men; PWID, people who inject drugs; TB, tuberculosis.

In the HIV study, 53.9% of participants on ART were receiving integrase inhibitors, while 36.9% and 8.6% were receiving non‐nucleoside reverse transcriptase inhibitors and protease inhibitors, respectively.

In the HIV/HCV study, 80.5% of 118 participants treated against HCV received an interferon‐free DAA regimen. Among the 253 (12.4%) participants with HCV genotype reported the most frequent were genotype 1 (55.3%) and 3 (41.5%). In 929 (45.5%) participants with known liver fibrosis stage, 83.6% had no or mild liver fibrosis (stage F0/F1), and 6.9% had cirrhosis (F4).

### 3.1 HIV CoC

Of 1028 participants in care, 1014 (98.6%, 95% confidence interval [CI] 97.7–99.3) were on ART, and 882 (87.0% of those on ART, 84.7–88.7) were virologically suppressed. A total of 98.2% PLHIV were on ART in Kyiv and 99.7% in Kyiv Region, while the percentage of suppressed was 85.7% in Kyiv and 90.0% in Kyiv Region.

In a sensitivity analysis in the 894 participants who had been on ART for >6 months, the overall proportion of virologically suppressed increased to 90.8%, with both sites above 90%.

### 3.2 HCV CoC

**Figure 1:**
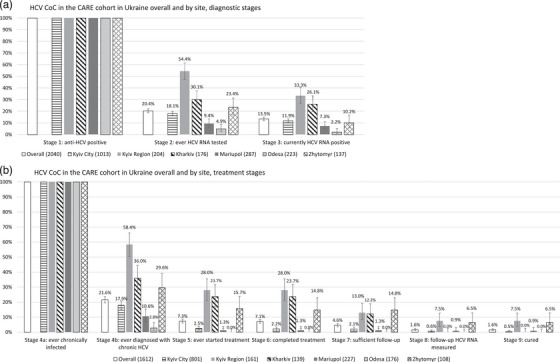
HCV CoC at enrolment in the CARE Cohort in Ukraine overall and by clinical site (error bars: 95% confidence intervals). The χ2 test provides evidence of significant difference between sites at all stages of the diagnostic and treatment cascade (*p*< 0.0001).

Of 2040 anti‐HCV‐positive PLHIV (Figure 1a), 20.4% (95% CI 18.7–22.3) were ever HCV‐RNA tested, and 13.5% (12.0–15.0) were currently HCV‐RNA positive at baseline. The lowest proportion of participants tested for HCV‐RNA and currently HCV‐RNA positive were in Odesa (4.9% and 2.2%), with the highest in Kyiv Region (54.4% and 33.3%).

Of an estimated 1612 ever chronically infected participants (Figure 1b), 21.6% (18.7–22.1) had ever been diagnosed before or at enrolment in CARE. Treatment had been started in 7.3% (6.1–8.7) of participants, with the highest proportions in Kyiv Region (28.0%) and Kharkiv (23.7%). A total of 7.1% of eligible participants had completed treatment, 4.6% had completed treatment >12/24 weeks prior to enrolment, and 1.6% had a post‐treatment HCV‐RNA measurement. HCV cure had been achieved in 1.6% of ever chronically infected (1.0–2.3) and ranged from 0% in Odesa to 7.5% in Kharkiv.

Additionally, 1.9% had a negative HCV‐RNA measurement after starting treatment but did not make our definition of HCV cure, as they had either not completed treatment, or treatment completion was too close to enrolment date, or the HCV‐RNA measurement was out of the window period specified.

### 3.3 HCV care facility survey

The proportion of HCV‐RNA tested exceeded 50% only in Kyiv Region, which was the only site with testing available for all anti‐HCV‐positive individuals for free. In two sites with the largest percentage of treated participants, Kyiv Region (28.0%) and Kharkiv (23.7%), DAA treatment was available for all. In the three sites reporting a limited amount of available DAA courses, the proportion of treated differed from 1.3% in Mariupol to 14.8% in Zhytomyr. No treatment was available in Odesa.

In Kyiv and Kharkiv, where PLHIV had to pay for an HCV‐RNA test after treatment completion, the proportion tested for HCV cure did not exceed 30%; however, it ranged from 43.8% to 66.4% in the sites providing free tests.

## 4 DISCUSSION

In this cross‐sectional study, we described the right‐hand side of the HIV CoC and a nine‐stage HCV CoC in PLHIV from Ukraine, at their CARE cohort enrolment visit between 1 January 2019 and 1 June 2020.

In the HIV study, 98.6% participants were on ART, and 86.4% were virally suppressed, which is lower than 94% reported in the national information bulletin in 2020 [26]. The higher HIV‐RNA cutoff in the report (1000 copies/ml, compared to 200 copies/ml in our study) might contribute to this difference. Nonetheless, this target was achieved among PLHIV receiving ART for >6 months at enrolment.

In the HIV/HCV study, the gap between the number of anti‐HCV positive and ever HCV‐RNA tested was the most prominent, with a range of 4.9%–54.4% participants having ever been tested across sites as of the enrolment date. According to the national report, among the regions included in our study, Kyiv had the lowest number of HCV cases (65.0 per 100,000 population) in 2018, while Kyiv Region had the highest equal to 178.1 per 100,000, which might be attributed to the diagnostic capacities [27]. In our study, over 50% of anti‐HCV‐positive persons had been tested for HCV‐RNA in the Kyiv Region only, where HCV‐RNA tests were provided for free. Notably, 13.5% of anti‐HCV‐positive persons (65.9% of those ever tested for HCV‐RNA) were RNA positive at baseline, mostly due to the lack of treatment.

The proportion of treated participants was low, with a striking heterogeneity across sites, and was strongly determined by medication availability. The large gap between the number of treated and the number with sufficient follow‐up in two sites with DAAs available for all suggests that treatment became available recently, explaining the low treatment coverage prior to enrolment.

The influence of predictors associated with HIV treatment outcomes [28] and HCV diagnosis and DAA treatment in other settings [29, 30, 31] requires further investigation in the CARE cohort, as well as the factors behind the striking difference in viral suppression between the HIV and HIV/HCV studies.

The key strength of our study is a large population of PLHIV enrolled from a country facing a major HIV and HCV epidemic. HIV/HCV study participants were enrolled from six regions of Ukraine, allowing for the evaluation of intra‐country variability, while HIV study participants donated blood, adding a host genetic dimension to the future analyses of HIV outcomes. The cohort structure will also allow for continuous follow‐up to monitor the changes in ART and DAA uptake over time. We successfully collected 2021 data (not shown), even though COVID‐19 remained a threat in Ukraine due to the low vaccination rate [32], causing 352 excess deaths per 100,000 in 2021 [33].

There are several limitations in this study. We could not investigate the proportion of people diagnosed with HIV, since all participants were linked to care. All HIV study participants donated blood, therefore, may be healthier due to better engagement in care. The HIV/HCV study enrolled coinfected participants from six regions of Ukraine, therefore, the results cannot be generalized to the monoinfected individuals, nor for the whole country. We were unable to capture reinfections since we used the first HCV‐RNA test 12/24 weeks after treatment completion to define the cure; further, people with negative HCV‐RNA tests after treatment not falling in the defined timeline were not considered cured. Finally, the overall indicators were driven by the site in Kyiv enrolling the largest number of participants, while site‐level CoC provided more granular information.

## 5 CONCLUSIONS

Our study demonstrated a high level of ART coverage in PLHIV in clinical care in the sites in Ukraine, while the last 90% target was achieved in participants receiving ART >6 months at enrolment. However, there is a long way to go towards HCV elimination. The CARE cohort has continued to collect data from the clinics in Ukraine during 2022 which will contribute to the aim of describing and addressing the gaps in HIV and HCV care occurring due to the war, with real‐life data. The findings of the present study may further serve as a baseline to assess the impact of the war on PLHIV in Ukraine.

## COMPETING INTERESTS

The authors of the manuscript have no competing interests to declare.

## AUTHORS’ CONTRIBUTIONS

OF, JR, LP and JL contributed to the study design and developing analysis plan. OF, JR and LP contributed to the analysis and interpretation of data. OF wrote the first draft of the manuscript and subsequent drafts after revisions. LP and JR reviewed all versions of the manuscript. IK, LH, AK, OS, NH, IKh, AN, VR, MK, TM, IP, MA, OV and OVal contributed to the recruitment of participants and coordination of the enrolment, reviewed the final version of the manuscript and contributed to the interpretation of the data.

## FUNDING

This publication is based upon work from CARE Consortium, which has received funding from the European Union's Horizon 2020 Research and Innovation programme (grant agreement No 825673).

## Data Availability

The data that support the findings of this study are available from the corresponding author upon reasonable request.
